# “Bilious” Attacks, or Acute Intestinal Auto-toxemia

**Published:** 1904-08

**Authors:** G. W. Tobias

**Affiliations:** New York


### "BILIOUS" ATTACKS, OR ACUTE INTESTINAL AUTO-TOXEMIA.

BY G. W. TOBIAS, M.D.

Luff, the London authority on gout, describes this disease as a "metabolic auto-toxemia, both intestinal and hepatic, which is usually followed by a deposit of sodium biurate in the joints and tissues." The individual predisposed to gout reacts differently to such various agencies as climate, food, etc.

Indigestion plays a greater role as a causative factor in the production of those symptoms attributed to excess of uric acid than is generally believed; urates, however, being merely an indication, and not the essential cause of gout. The lithemic manifestations so often attributed to disturbance of the nervous system, are primarily due to interference with normal digestion; or, rather, the nervous strain, worry and anxiety from any cause gives rise to indigestion, and in turn to gouty manifestations in some form. We have all noted attacks of gout, migraine, hay asthma, or some of the protena phases of disease betrayed by the presence of an excess of uric acid, coming on after an attack of acute indigestion.

A large class of patients are troubled more or less frequently with a migraine and intestinal auto-intoxication which is not of necessity followed or preceded by gouty deposits in the articulations, although such attacks are followed by heavy deposits of urates and phosphates in the urine, these deposits being the measure of the destructive metabolism of the nucleins of the body cells, chiefly of the leukocytes, in response to the invasions of poisons or toxines. As most of the toxines setting up this destructive metabolism and consequent uric acid production are of intestinal origin, diet in gout should be regulated solely with regard to the diminution of intestinal fermentation and putrefaction.

This form of the gouty manifestation is especially frequent with highly nervous and very active mental workers, and also amongst certain women. It usually occurs after undue excitement, worry, or excesses of the table, especially if the food contents of the stomach are much diluted with certain kinds of malt liquors or wines. Lager beer, however, I find generally tolerated, and even beneficial to this class of patients.

Usually the premonitory symptoms of a "bilious attack" are felt on rising in the morning; there is great lassitude, muscular relaxation, want of enthusiasm; a heavy, stiff feeling of the eyeballs, and as the day advances, nausea, severe migraine, culminating in complete exhaustion, vomiting, slow pulse, cold extremities, etc.

The role of the liver in this auto-toxemia is a negative one, being inability to perform its chief normal function as a "poison filter" and to absorb or transform into harmless excretory substances the excess of toxines brought to it by the portal vein.

The paroxysms of pain in the head in some cases are so violent that something has to be attempted to give immediate relief, but hitherto I have found purgatives, effervescing sedatives, antipyretics, hypnotics, anodynes and drugs such as colchicine, citrate of potash, etc., ineffective to arrest these conditions until the gradual elimination of the toxines takes place, which requires from twelve to twenty-four hours. One of the great difficulties in these cases is that remedies are usually vomited immediately. I have recently been most successful with colchi-sal capsules in two very severe cases which had previously baffled all my efforts to give prompt relief during similar attacks.

(1) Mrs. P., highly nervous temperament, age 26, suffers periodically from this form of auto-toxemia, generally immediately before the menses. She is a high liver, and in spite of warnings imbibes freely at times. Previously I had never been able to relieve her, but during the last two attacks I have been able to do so promptly by giving her one capsule (20 centigrams) of colchi-sal every ten minutes until six capsules were taken, by which time she fell asleep, awaking two hours later, completely free from migraine and all symptoms. This took place on both occasions.

(2) Mr. S. F., nervous temperament, age 46, has suffered since boyhood from very acute attacks of "bilious toxemia" or migraine, at which time he became almost insane with pain—so much so that the neighbors at such times became alarmed at his cries. The feeling of nausea and weariness, pain in the calves of the legs, always preceded by several hours the acute symptoms of headache and vomiting. The attacks come on apparently from no special cause and occur sometimes twice a month, sometimes once only in three months. The patient is usually able to detect the premonitory symptoms of an attack the evening before, and then abstains from food, so that the next day vomiting only brings up some clear mucous fluid. Calomel administered at night and saline purgatives in the morning never yet succeeded in preventing the usual symptoms following in succession. Diet does not seem to inhibit these attacks. The auto-toxemia in this patient is most severe, and the same conditions are repeated on each occasion. The heart's action is reduced to under fifty pulsations, the extremities become cold, even in summer, and the patient seems to be able to endure the pain only by sitting up in bed with the head held back so that the cords of the neck are violently strained; the breath is usually very acrid and peculiar in odor; while the urine becomes almost alkaline and highly charged with phosphates and urates for forty-eight hours after each attack.

I was consulted during his last attack and succeeded in arresting the headache within twenty minutes of his taking five capsules of colchi-sal (each capsule at intervals of five minutes), when he fell into a deep sleep and awoke the next morning perfectly well and refreshed, although the intensity of the paroxysms and excitement caused thereby, usually made him a wreck the next day. I have since found this treatment universally successful wherever the capsules of colchi-sal are retained a sufficient length of time to allow of their contents being absorbed.—*New York, April 15, 1904.*

